# Vestibular Infant Screening (VIS)–Flanders: results after 1.5 years of vestibular screening in hearing-impaired children

**DOI:** 10.1038/s41598-020-78049-z

**Published:** 2020-12-03

**Authors:** Sarie Martens, Ingeborg Dhooge, Cleo Dhondt, Saartje Vanaudenaerde, Marieke Sucaet, Lotte Rombaut, An Boudewyns, Christian Desloovere, Sebastien Janssens de Varebeke, Anne-Sophie Vinck, Robby Vanspauwen, Dominique Verschueren, Ina Foulon, Charlotte Staelens, Karen Van den Broeck, Claudia De Valck, Naima Deggouj, Nele Lemkens, Lisa Haverbeke, Mieke De Bock, Okan Öz, Frank Declau, Benoit Devroede, Christoph Verhoye, Leen Maes

**Affiliations:** 1 Faculty of Medicine and Health Sciences, Department of Rehabilitation Sciences, Ghent University, 9000 Ghent, Belgium; 2 Department of Oto-Rhino-Laryngology, Ghent University Hospital, 9000 Ghent, Belgium; 3 Faculty of Medicine and Health Sciences, Department of Head and Skin, Ghent University, 9000 Ghent, Belgium; 4 Department of Otorhinolaryngology, Head and Neck Surgery, Antwerp University Hospital, University of Antwerp, 2650 Edegem, Belgium; 5 Department of Otorhinolaryngology, Head and Neck Surgery, University Hospitals Leuven, 3000 Leuven, Belgium; 6 Department of Otorhinolaryngology, Head and Neck Surgery, Jessa Hospital, 3500 Hasselt, Belgium; 7 Department of Otorhinolaryngology, Head and Neck Surgery, AZ Sint-Jan Hospital Bruges, 8000 Bruges, Belgium; 8 European Institute for Otorhinolaryngology, Head and Neck Surgery, Wilrijk, 2610 Antwerp, Belgium; 9 Department of Otorhinolaryngology, Head and Neck Surgery, AZ Maria Middelares Hospital Ghent, 9000 Ghent, Belgium; 10 Department of Otorhinolaryngology, Head and Neck Surgery, University Hospital Brussels, 1090 Brussels, Belgium; 11 Department of Otorhinolaryngology, Head and Neck Surgery, AZ Delta Hospitals, 8930 Menen, Belgium; 12 Centre for Ambulatory Rehabilitation, Stappie VZW, 8400 Ostend, Belgium; 13 Department of Otorhinolaryngology, Head and Neck Surgery, AZ Turnhout Hospital, 2300 Turnhout, Belgium; 14 Institute of Neurosciences and Department of Oto-Rhino-Laryngology and Head and Neck Surgery, Université Catholique de Louvain, 1200 Brussels, Belgium; 15 Department of Otorhinolaryngology, Head and Neck Surgery, ZOL Genk Hospital, 3600 Genk, Belgium; 16 Department of Otorhinolaryngology, Head and Neck Surgery, ASZ Aalst Hospital, 9300 Aalst, Belgium; 17 Department of Early Counseling and Audiology, Centre for Ambulatory Rehabilitation Sint-Lievenspoort, 9000 Ghent, Belgium; 18 The Eargroup, 2100 Antwerp-Deurne, Belgium; 19 Department of Otorhinolaryngology, Head and Neck Surgery, GZA Hospitals Sint-Vincentius, 2018 Antwerp, Belgium; 20 Department of Otorhinolaryngology, Head and Neck Surgery, Queen Fabiola Children’s University Hospital, 1020 Brussels, Belgium; 21 Department of Otorhinolaryngology, Head and Neck Surgery, AZ Sint-Lucas Hospital, 8310 Bruges, Belgium

**Keywords:** Population screening, Paediatric research

## Abstract

Due to the close anatomical relationship between the auditory and vestibular end organs, hearing-impaired children have a higher risk for vestibular dysfunction, which can affect their (motor) development. Unfortunately, vestibular dysfunction often goes unnoticed, as vestibular assessment in these children is not standard of care nowadays. To timely detect vestibular dysfunction, the Vestibular Infant Screening–Flanders (VIS–Flanders) project has implemented a basic vestibular screening test for hearing-impaired infants in Flanders (Belgium) with a participation rate of 86.7% during the first year and a half. The cervical Vestibular Evoked Myogenic Potentials (cVEMP) test was applied as vestibular screening tool to map the occurrence of vestibular (mainly saccular) dysfunction in this population. At the age of 6 months, 184 infants were screened. No refers on vestibular screening were observed in infants with permanent conductive hearing loss. In infants with permanent sensorineural hearing loss, a cVEMP refer rate of 9.5% was observed. Failure was significantly more common in infants with severe-profound compared to those with mild-moderate sensorineural hearing loss (risk ratio = 9.8). Since this is the first regional study with a large sample size and successful participation rate, the VIS–Flanders project aims to set an example for other regions worldwide.

## Introduction

The vestibular end organs play an essential role in the maintenance of balance control and gaze stabilisation during posture and movements^1^. Several studies in young children with a bilateral severe vestibular dysfunction have shown a reduced balance performance and a delayed acquisition of gross motor milestones (e.g. head stabilisation, sitting and independent walking)^1–10^. Vestibular dysfunction can also influence fine motor skills, as well as writing, reading and learning skills, and may even hamper the cognitive and socio-emotional development of children^1,11–17^. Timely detection of vestibular dysfunction is crucial in order to provide parental counselling and start adequate vestibular rehabilitation at a young age^1,18,19^. Therefore, a standard vestibular assessment is needed in young children that are at risk for a vestibular dysfunction, such as hearing-impaired children. Because of the close anatomical and embryological relationship between the peripheral auditory and vestibular organs, it seems evident that whatever reason causes damage to the auditory structures, may also negatively affect the vestibular end organs^8,20^. This was confirmed in a systematic review by Verbecque et al. who demonstrated a significantly higher occurrence of vestibular dysfunction in children with sensorineural hearing loss compared to normal-hearing children in all included studies^21^.

**Figure 1:**
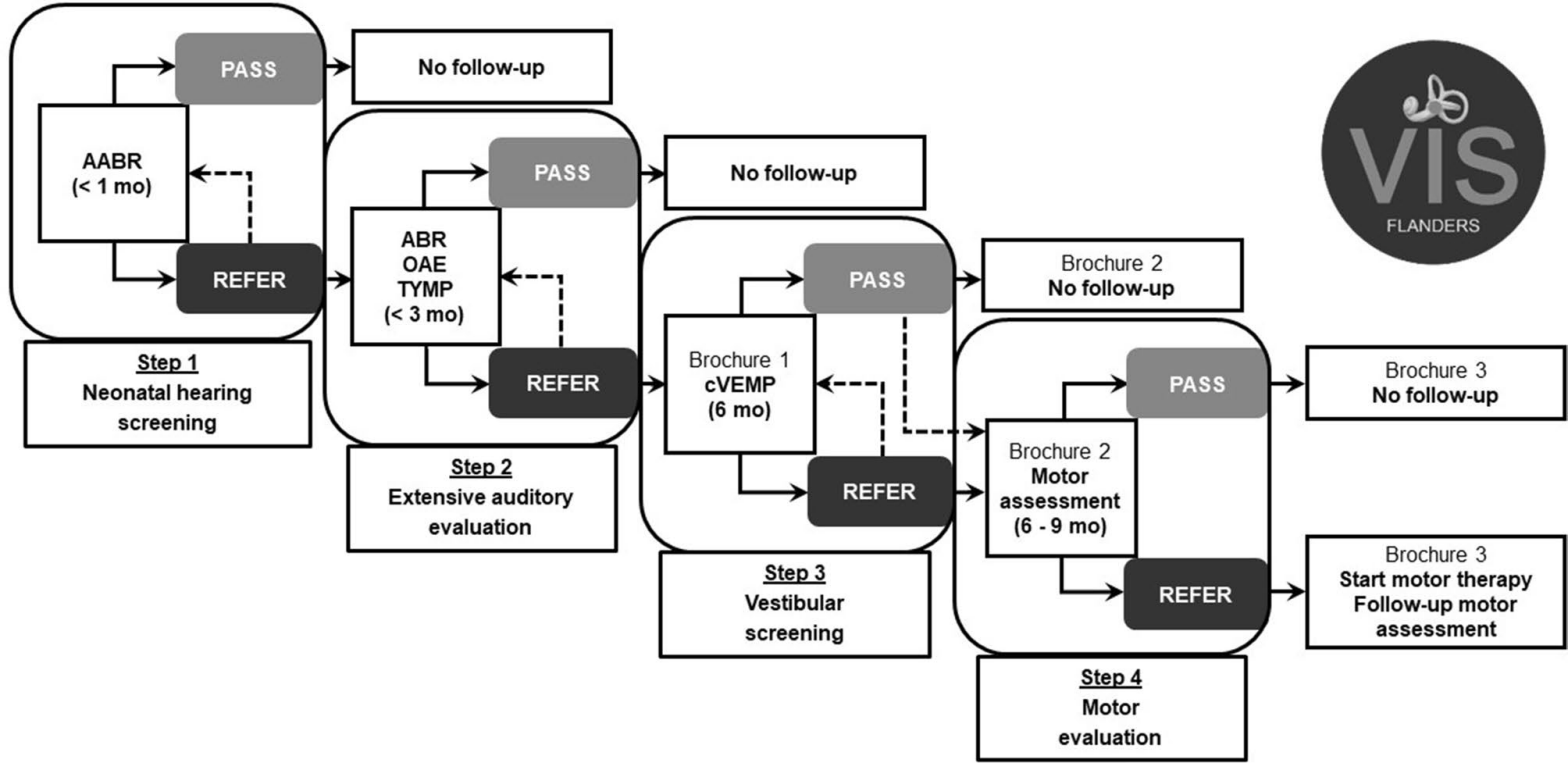
The existing neonatal hearing screening protocol in Flanders (Belgium) in addition with the vestibular screening protocol. Additional information about the brochures for parents: Brochure 1 = ‘Vestibular screening VIS–Flanders’; Brochure 2 = ‘What after the vestibular screening?’; Brochure 3 = ‘Tips and tricks for vestibular dysfunction’. *(A)ABR* (automated) auditory brainstem responses, *OAE* otoacoustic emissions, *TYMP* tympanometry, *cVEMP* cervical Vestibular Evoked Myogenic Potentials, *VIS–Flanders* Vestibular Infant Screening–Flanders.

Recently, there is an increasing interest among clinicians in the vestibular function of hearing-impaired children and several researchers have proven the feasibility of vestibular testing from an early age on by adapting the standard test protocol to children^22–25^. However, paediatric vestibular dysfunction often goes unnoticed due to the atypical expression of vestibular symptoms and the limited communicative abilities of young children^18,26^. Additionally, paediatric vestibular assessment remains challenging and time-consuming, and child-friendly equipment and two experienced examiners are often needed. Therefore, paediatric vestibular assessment in clinical practice is only performed in specialized centres that have appropriate equipment, and is mostly limited to cochlear implant candidates^27–31^ and children with a suspicion of vestibular dysfunction^32^. To overcome the lack of vestibular assessment in young hearing-impaired children, the Vestibular Infant Screening–Flanders (VIS–Flanders) project has been set up in June 2018 in Flanders (Belgium) in order to give each newborn with a confirmed hearing loss access to a basic screening of the vestibular function by using only one vestibular test. This Flemish research project includes a standard vestibular screening by means of the cervical Vestibular Evoked Myogenic Potentials (cVEMP) test at the age of 6 months^33^. Although the vestibular screening by means of the cVEMP mainly evaluates saccular function and does not evaluate all vestibular end organs, the cVEMP is chosen as screening tool because this is a short, objective and child-friendly test^2,23,34,35^. Several studies have also demonstrated a higher percentage of otolith dysfunction compared to semicircular canal dysfunction in hearing-impaired children due to the closer anatomical and embryological relationship between the otolith organs and the cochlea^36–38^. Furthermore, the results of the cVEMP strongly correlate with the motor performance of hearing-impaired children, as the otoliths detect linear and gravitational accelerations during translational movements^2,3,10^. The rationale for vestibular screening at the age of 6 months is the following: (1) the hearing loss is confirmed in the majority of the children by that age^39^, (2) gross motor milestones can already give an idea of the child’s vestibular function^3^, and (3) most children have already developed sufficient head stabilisation due to sufficient development and control of neck musculature, which is fundamental for reliable cVEMP assessment^40^. In addition, possible cochlear implant surgery which entails a potential risk for vestibular (mainly otolith) deficits^31,41–43^, has not been performed yet^44^. As a result, the vestibular (mainly saccular) status is known before some of these children will undergo cochlear implant surgery. This early screening enables a timely referral to limit the impact on the child’s motor development^19^. The rationale of implementing this screening specifically for hearing-impaired children is their higher risk for vestibular dysfunction as mentioned earlier. Moreover, the vestibular screening could be perfectly added to the existing neonatal hearing screening programme for all newborns in Flanders (Belgium) (Fig. 1), which covers 96.9% of the target population^45^. Infants who fail the hearing screening twice, are referred within the first weeks of life for a subsequent extensive auditory evaluation in a limited number of specialized centres (i.e. reference centres)^46^. Since all reference centres have auditory brainstem responses (ABR) equipment, which includes a cVEMP module, the implementation of the vestibular screening is practically feasible on a large scale. After confirmation of the hearing loss, the vestibular screening is scheduled at the age of 6 months in one of the reference centres (Fig. 1). So far, only one pilot study applied the cVEMP as a vestibular screening tool in infants in the context of the neonatal hearing screening programme^47^. This study of Verrecchia et al. showed a good feasibility when using the cVEMP as a vestibular screening tool in infants. However, the latter study only included a small number of infants screened at very young age (mean age: 2.3 months) and included both normal-hearing and hearing-impaired infants.

The aim of this paper is to report the vestibular screening results obtained in the first cohort of hearing-impaired infants enrolled in the VIS–Flanders programme and to increase awareness about vestibular dysfunction in these children. Therefore, this is the first study using the cVEMP as a standard vestibular screening tool on a large regional scale for hearing-impaired infants, regardless of their degree and type of hearing loss. These results will enable a more accurate and representative estimation of the occurrence of vestibular (mainly saccular) dysfunction in hearing-impaired infants.

## Methods

### Subjects

All infants with a confirmed permanent hearing loss (regardless of the laterality, degree and type of hearing loss) detected by the neonatal hearing screening programme were screened for a possible vestibular dysfunction in one of the 25 reference centres involved in the hearing screening programme. Infants with early-onset permanent hearing loss (before 10 months of age) (e.g. post-bacterial meningitis) were also included in the study. To determine the hearing loss characteristics, the results of the click-evoked ABR, high frequency tympanometry (1000 Hz), and transient evoked or distortion product otoacoustic emissions (OAE) were collected across all centres. The degree of hearing loss was categorized in accordance with the criteria of the International Bureau for Audiophonology. In subjects with bilateral hearing loss, the worst ear was taken into account to categorize the degree of hearing loss. The leading ethical committee of the Ghent University Hospital and all local ethical committees of the participating centres approved data collection of all subjects (registration number B670201835971). According to the Helsinki Declaration, written informed consents of parents were obtained before the start of the vestibular screening test.

### Vestibular screening protocol

Around the age of 6 months, hearing-impaired infants were screened with the cVEMP test, as depicted in the previous publication of Martens et al.^33^. To circumvent frequently occurring middle ear disorders in infants, bone-conducted stimuli (RadioEar B71W, Middelfart, Denmark) were presented at the ipsilateral mastoid^40,48^. More specific, 500 Hz tone bursts were used with a rise/fall time of 1 ms and a 2 ms plateau time, an intensity level of 59 decibel normalized hearing level (dB nHL) (i.e. equivalent to 129 dB force level (FL)), and a stimulus repetition rate of 5 Hz. In all participating centres, the Neuro-Audio commercial device and accompanying software (Neurosoft, version 2010, Ivanovo, Russia) was used to automatically record and visually monitor electromyographic (EMG) background activity on a screen. The electromyographic (EMG) activity was measured by placing 1 ground electrode on the forehead, 1 reference (i.e. inverting) electrode on the sternum (i.e. between 1 and 2 cm below the sternoclavicular junction) and 2 active (i.e. noninverting) electrodes symmetrically on both bellies of the sternocleidomastoid (SCM) muscles. Subsequently, all participating centres were instructed to apply impedances of the self-adhesive electrodes below 5 kΩ and inter-electrode impedances below 2 kΩ before performing the vestibular screening test. The algorithm of the software automatically applied a pre-stimulus EMG recording of at least 20 ms to quantify the EMG background activity. Data acquisition was automatically accepted if the mean rectified voltage (MRV) values ranged between 80 and 250 µV and rejected outside this range. The EMG signals underwent amplification (5000 times) and bandpass filtering (10–1500 Hz). Minimum 35 and maximum 100 accepted sweeps were recommended and averaged for each trial.

One examiner operated the software, while the second examiner kept the child and the bone conductor in place during monaural stimulation. During the screening, the subjects were placed with the upper body upon a sloping pillow in supine position. Thereby, the examiner rotated and raised the head towards the contralateral (non-test) side and placed the bone conductor with the index finger at the ipsilateral (test) mastoid without touching the helix of the auricle, while slightly supporting the head with the other hand. During each acquisition, special attention was paid to ensure that the bone conductor was always correctly kept in the above described position. At the same time, the parent provided distraction at the contralateral side with some toys or a movie^23,33^. In this way, the child was motivated to maintain sufficient and stable SCM muscle contraction. Both examiners could observe the automatically EMG controlled recording on the screen in order to compare SCM muscle tension within and between the different trials of both sides. At least two trials were recorded on each side to check the reproducibility of the waveform. If more than two reproducible trials were recorded, two trials with comparable SCM muscle tension (i.e. preferred difference in averaged EMG levels ≤ 30 µV) were selected for further analysis on each side. To assure that all centres applied the same procedure and that reliable cVEMP results were obtained, one audiologist of the VIS–Flanders team was responsible to train and support all centres in performing the screening.

### Referral criteria

cVEMP responses are considered as normal (i.e. pass) if at least two reproducible biphasic P1-N1 waveforms are observed. Additionally, the rectified amplitude of at least one of the trials needs to reach the normative cut-off value of 1.3, which is based on normative data of the Ghent University Hospital obtained in a group of 34 healthy control subjects (mean age = 7.6 months; SD = 1.5 months). cVEMP responses are considered as aberrant (i.e. refer) in case of inconclusive (i.e. insufficient SCM muscle tension), abnormal (i.e. reproducible waveforms but rectified amplitude < 1.3 for all trials) or absent (i.e. no reproducible waveforms) responses.

Figure 1 offers an overview of the existing neonatal hearing screening protocol in Flanders (Belgium) in addition with the vestibular screening protocol and subsequent referral for motor assessment if needed. If a bilateral pass is obtained (after the first or, in case of a retest, second vestibular screening), an information brochure is provided and the screening results are discussed with the parents or caregivers along with the limitations of the screening test (i.e. only testing otolith (mainly saccular) function and the possibility of late-onset vestibular dysfunction). No further vestibular or motor follow-up is provided, unless parents or caregivers are concerned about the child’s motor development. If a unilateral or bilateral refer is observed during the first screening, a retest is performed before the age of 10 months (i.e. within 3 months after the first screening) in order to confirm the results (Fig. 1). In case there is no need for the treating otorhinolaryngologist to repeat the screening, as the results are reliable and it is practically not feasible to repeat the screening (e.g. due to limited time before cochlear implantation or a difficult home situation of the child), the results of the first screening are taken into account. Therefore, the results of the final screening test are reported (unless stated otherwise). Children with a unilateral or bilateral refer during the final screening fail the vestibular screening test. Subsequently, those children are referred for motor assessment to evaluate the necessity of physiotherapy (after the parents have received appropriate counselling and an information brochure)^33^.

### Statistical analysis

The Statistical Package for Social Science (SPSS) software (IBM, version 26.0, Armonk, NY) was applied for data analysis. Descriptive statistics were used to compare the screening results among different groups regarding the degree, laterality and type of hearing loss. The two-tailed Fisher’s Exact test was used in infants with sensorineural hearing loss to evaluate the association between the screening results (i.e. bilateral pass, unilateral or bilateral refer), the laterality of sensorineural hearing loss (i.e. unilateral, bilateral) and the degree of sensorineural hearing loss (i.e. unilateral or bilateral mild-moderate, unilateral or bilateral severe-profound). The latter associations were considered as statistically significant if *p* < 0.05.

## Results

### Subjects

From June 2018 until February 2020, vestibular screening was offered to 191 infants. Four of these infants were excluded from the dataset because further audiological assessment showed normal hearing. In one case, the parents withdrew their consent. Moreover, two additional cases were excluded as the first vestibular screening results were inconclusive (i.e. insufficient SCM muscle tension) and the parents refused the second screening test. The vestibular screening results of 184 infants (90 boys, 94 girls) were included in the analysis. At the moment of the first screening, the mean age of the infants was 6.8 months (standard deviation (SD): 1.8 months). 8.7% (16/184) of these infants were rescheduled for a second vestibular screening (8 boys, 8 girls). During the second screening, the infants had a mean age of 9.3 months (SD: 2.2 months). None of the infants received cochlear implants before the first or second screening. A unilateral or bilateral permanent sensorineural hearing loss was reported in the majority of the subjects (91.8% (169/184)), whereas a unilateral or bilateral permanent conductive hearing loss (e.g. due to external auditory canal atresia) was less frequently found (8.2% (15/184)). None of the children had a mixed type of hearing loss. A congenital hearing loss was found in 98.9% (182/184), whereas an early-onset hearing loss (i.e. before the age of 10 months) was reported in 1.1% (2/184) of the subjects. Both infants had sensorineural hearing loss caused by meningitis at the respective age of 2 months and 4 months.

### Participation rate

The Flemish infant welfare agency ‘Kind en Gezin’ reported a permanent congenital hearing loss (including all types of hearing loss) in 122 infants on average per year, which is based on the most recently available data (i.e. between 2013 and 2016)^45^. Consequently, the percentage of the target population with a congenital hearing loss subjected to the vestibular screening during the first 21 months of this project was estimated at 86.7% (182/210) (i.e. participation rate).

### Refer rate

All infants with a permanent conductive hearing loss (n = 15) finally passed the vestibular screening test on both sides. More specifically, bilateral pass was found in 93.3% (14/15) on the first vestibular screening test, whereas 6.7% (1/15) needed a retest due to insufficient SCM muscle tension. Only infants with permanent sensorineural hearing loss (i.e. congenital and early-onset) were taken into account for further analysis. In infants with sensorineural hearing loss (n = 169), bilateral pass was found in 90.5% (153/169) of the infants tested, yielding a refer rate of 9.5% (16/169). Table 1 provides an overview of the vestibular screening results in all infants with sensorineural hearing loss. Unilateral or bilateral abnormal or absent responses were recorded in 7.7% (13/169) of the infants tested and inconclusive results were obtained in 1.8% (3/169) due to insufficient SCM muscle tension. An overview of the patients with a unilateral or bilateral refer at the first or second vestibular screening is provided in Table 2, including additional information about the hearing loss characteristics and the vestibular screening results.

**Table 1:** Overview of the vestibular screening results in 169 infants with sensorineural hearing loss.

1st screening result	2nd screening result	Final screening result
Pass (Bilat.) 88.8% (150/169)	N/A 88.8% (150/169)	Pass (Bilat.) 90.5% (153/169)
Refer (Unilat./Bilat.)—inconclusive 6.5% (11/169)	Pass (Bilat.) 1.8% (3/169)	
	Refer (Unilat./Bilat.)—inconclusive 1.8% (3/169)	Refer (Unilat./Bilat.)—inconclusive 1.8% (3/169)
	Refer (Unilat./Bilat.)—abnormal or absent 3.0% (5/169)	Refer (Unilat./Bilat.)—abnormal or absent 7.7% (13/169)
Refer (Unilat./Bilat.)—abnormal or absent 4.7% (8/169)	Refer (Unilat./Bilat.)—abnormal or absent 2.4% (4/169)	
	N/A* 2.4% (4/169)	

*The treating otorhinolaryngologists and audiologists agreed on settling for only one screening, as it was impossible to reschedule the second vestibular screening before cochlear implantation (n = 3) or due to a problematic home situation (n = 1).

*Bilat.* Bilateral, *Unilat.* Unilateral, *N/A* not applicable.

**Table 2:** Overview of all infants with sensorineural hearing loss with a unilateral or bilateral refer on the first or second vestibular screening (n = 19).

HL laterality	HL degree	Etiology	1st screening result	2nd screening result
Unilat.	Profound right	Aplasia n. VIII right	Refer (Unilat.)—absent right	Refer (Unilat.)—absent right
Bilat.	Profound	cCMV	Refer (Unilat.)—absent right	N/A
Unilat.	Severe left	Unknown	Refer (Bilat.)—inconclusive	Refer (Unilat.)—absent left
Unilat.	Profound left	Aplasia n. VIII left	Refer (Unilat.)—absent left	Refer (Unilat.)—absent left
Bilat.	Profound	Unknown	Refer (Bilat.)—inconclusive	Refer (Bilat.)—abnormal
Bilat.	Profound	DFNB1	Refer (Bilat.)—absent	N/A
Unilat.	Moderate left	Meningitis	Refer (Unilat.)—absent left	Refer (Unilat.)—absent left
Unilat.	Profound right	cCMV	Refer (Bilat.)—inconclusive	Refer (Unilat.)—absent right
Bilat.	Moderate right—profound left	Unknown	Refer (Unilat.)—abnormal left	Refer (Unilat.)—absent left
Bilat.	Mild left—profound right	Feingold II	Refer (Bilat.)—inconclusive	Refer (Unilat.)—abnormal right
Bilat.	Moderate right—profound left	Meningitis	Refer (Bilat.)—inconclusive	Refer (Unilat.)—abnormal right
Unilat.	Severe right	Fetal hypoxia	Refer (Unilat.)—absent right	N/A
Bilat.	Profound	Usher I	Refer (Bilat.)—absent	N/A
Bilat.	Profound	CHARGE	Refer (Bilat.)—inconclusive	Refer (Bilat.)—inconclusive
Bilat.	Profound	DFNB35	Refer (Bilat.)—inconclusive	Refer (Bilat.)—inconclusive
Bilat.	Profound	Unknown	Refer (Bilat.)—inconclusive	Refer (Bilat.)—inconclusive
Unilat.	Moderate right	Unknown	Refer (Unilat.)—inconclusive left	Pass
Bilat.	Moderate	DFNB3	Refer (Bilat.)—inconclusive	Pass
Bilat.	Profound	DFNB1	Refer (Bilat.)—inconclusive	Pass

*HL* hearing loss, *Unilat.* unilateral, *Bilat.* Bilateral, *cCMV* congenital cytomegalovirus, *Feingold II* Feingold syndrome type 2, *Usher I* Usher Syndrome type 1, *CHARGE* coloboma of the eyes, heart defects, choanal atresia, growth and developmental retardation, ear abnormalities and deafness, *DFNB* autosomal recessive deafness, *N/A* not applicable.

### Relation with the laterality and degree of sensorineural hearing loss

**Figure 2:**
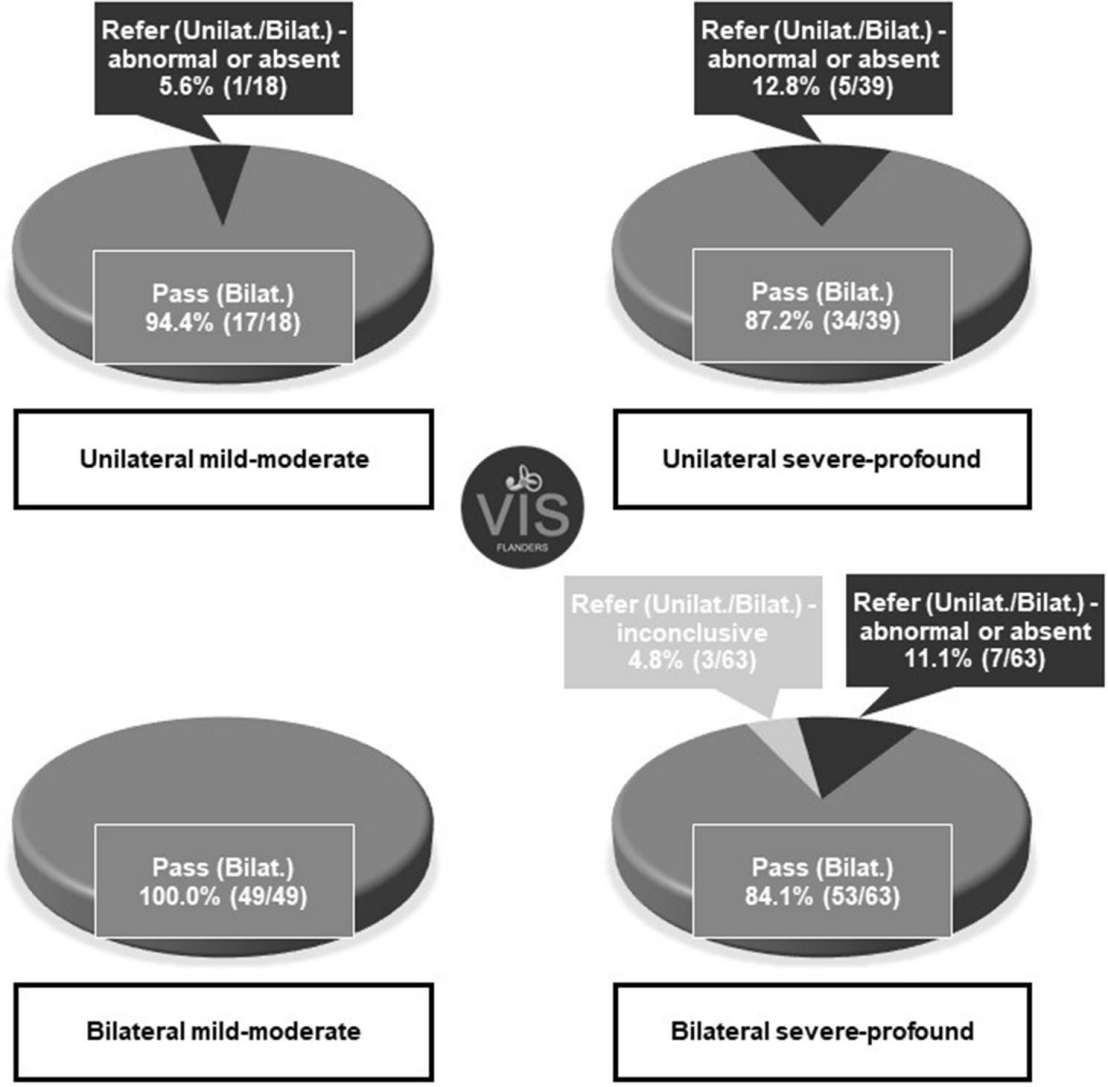
Vestibular screening results according to the degree and laterality of sensorineural hearing loss. *cVEMP* cervical Vestibular Evoked Myogenic Potentials, *VIS–Flanders* Vestibular Infant Screening–Flanders.

An overview of the laterality and degree of sensorineural hearing loss is given in Table 3. No significant difference could be observed between the refer rate of infants with a unilateral (10.5%, 6/57) or bilateral (8.9%, 10/112) hearing loss (*p* = 0.784). Unilateral absent cVEMP responses were found at the same side of the hearing loss in all six infants with a unilateral hearing loss who failed the screening (Table 2). In bilaterally hearing-impaired infants with abnormal or absent responses, the cVEMP results were more diverse (i.e. unilateral abnormal (n = 2), unilateral absent (n = 2), bilateral abnormal (n = 1) and bilateral absent (n = 2)). Concerning the degree of hearing loss, the results showed that a unilateral or bilateral refer occurred significantly more in infants with a unilateral or bilateral severe-profound hearing loss (14.7%, 15/102, 95% CI [9.1–22.9%]), compared to infants with a unilateral or bilateral mild-moderate hearing loss (1.5%, 1/67, 95% CI [0.3–8.0%]) (*p* = 0.003). Noteworthy, only one subject with a mild-moderate hearing loss failed the vestibular screening (Table 2). The calculated risk ratio (RR = 9.8) estimated that infants with a unilateral or bilateral severe-profound hearing loss have a higher chance to exhibit a unilateral or bilateral refer on the vestibular screening compared to infants with a unilateral or bilateral mild-moderate hearing loss. Figure 2 displays the vestibular screening results among the different groups of infants according to their degree and laterality of sensorineural hearing loss.

**Table 3:** Overview of the laterality and degree of sensorineural hearing loss in 169 infants.

Hearing loss	Mild-moderate	Severe-profound	Total (laterality)
Bilateral	29.0% (49/169)	37.3% (63/169)^a^	66.3% (112/169)
Unilateral	10.6% (18/169)	23.1% (39/169)	33.7% (57/169)
Total (degree)	39.6% (67/169)	60.4% (102/169)	

^a^15 of the 63 subjects who were classified with bilateral severe-profound hearing loss showed mild-moderate hearing loss in the other ear.

## Discussion

To our knowledge, this is the first large-scale regional study that aimed to accurately estimate the occurrence of vestibular (mainly saccular) dysfunction based on the cVEMP screening results of a representative group of hearing-impaired infants. Secondly, this study investigated whether the vestibular screening results differed among various hearing loss characteristics, including the type, degree and laterality.

In this study, 86.7% of the estimated target population participated to the vestibular screening. This finding demonstrates that it is indeed feasible to implement a vestibular screening test, covering the majority of congenitally hearing-impaired infants across an entire region. Nevertheless, an estimated 13.3% of the infants probably missed the vestibular screening, which may be attributed to parents who declined their child’s participation, subjects who did not show up at several appointments, subjects who moved abroad after birth, and critically ill infants in which auditory and vestibular evaluations were not a number one priority during the first year of life. Whereas a rather high participation rate was observed in our study, Verrecchia et al. (2019) reported a participation rate of only 60% in their target group. The authors of the latter study attributed the low participation rate to the preoccupation of the parents with the outcome of the hearing tests^47^. A number of other reasons could account for the different participation rate of both studies, such as the different ages of the study participants. Verrecchia et al. (2019) conducted the cVEMP test in younger infants (mean age: 2.3 months; SD: 1.9 months) compared to the VIS–Flanders project (mean age first cVEMP: 6.8 months; SD: 1.8 months). Moreover, the VIS–Flanders project solely focussed on infants with a confirmed hearing loss, whereas Verrecchia et al. (2019) also included normal-hearing infants at risk for hearing impairment. The higher participation rate in the current study may also be explained by the strong collaboration between the VIS–Flanders project and the meticulously organised neonatal hearing screening programme in Flanders, which enabled a large-scale implementation for the entire target population. The above mentioned differences emphasize the added value of implementing the cVEMP as a standard screening tool after the confirmation of the hearing loss around the age of 6 months. By then, parents are probably less likely to refuse the cVEMP test because some time is given first to emotionally process the child’s hearing loss, to start the child’s auditory rehabilitation, and to be properly informed about the importance of vestibular screening.

In the current study, all cVEMP refers were recorded in infants with a sensorineural hearing loss, as none of the infants with a permanent conductive hearing loss failed the vestibular screening. These findings were in accordance with the results of Sheykholesami et al.^49,50^, which indicated that the anatomical proximity of the cochlea and the vestibular end organs played a major role in the higher risk for vestibular dysfunction in sensorineural hearing-impaired infants. All children with a permanent conductive hearing loss in the latter study showed normal bone-conduction cVEMP responses^49^. Therefore, it is assumed that otolith (mainly saccular) dysfunction is less likely to occur in children with a permanent conductive hearing loss. In infants with sensorineural hearing loss, a refer rate of 9.5% was found in the current study, consisting of 7.7% of infants with unilateral or bilateral abnormal or absent cVEMP results and 1.8% of infants with inconclusive results. The presence of vestibular (mainly saccular) dysfunction remained unknown in the infants with inconclusive results caused by bilateral insufficient SCM muscle tension. Noteworthy, these infants were also classified in the refer group, because the lack of sufficient head stabilisation suggests the presence of a vestibular (otolith) dysfunction or a delayed motor development^5^. This implies that this specific group definitely needs referral for motor assessment. In contrast to our findings, the systematic review of Verbecque et al. reported a considerably higher percentage of deviant cVEMP results (i.e. 43.0% in general) on at least one side in children with sensorineural hearing loss^21^. This higher percentage can be explained by the fact that the included study populations in this systematic review mainly consisted of older children, which can result in different cut-off values, delayed-onset vestibular dysfunction and potential post-cochlear implant effects^27,28,30,31^. Moreover, most of the included studies focussed on children with severe-profound hearing loss^21^, whereas the current study also included children with mild-moderate hearing loss. Nevertheless, the cVEMP refer rate of 14.7% in children with severe-profound hearing loss in the current study is still lower compared to the included studies by Verbecque et al.^21^. However, these included studies were conducted in specialized centres for cochlear implantation, congenital cytomegalovirus infection, and complex pathologies, thus, possibly included subjects with a higher risk for vestibular dysfunction. While the majority of the included studies applied the cVEMP with air-conduction stimuli^21^, the current study used bone-conduction stimuli. The use of different stimuli may also explain different percentages of aberrant cVEMP results. Although saccular afferents predominantly project to cervical muscles, bone-conduction and air-conduction cVEMP tests stimulate the saccular macula with different modalities^51–53^. In other words, the mechanisms of activating otolithic receptors by air-conduction and bone-conduction stimuli are dissimilar but the exact mechanisms are still being investigated^54–56^.Moreover, the VIS–Flanders project is the first to offer the vestibular screening to all hearing-impaired infants in an entire region, which enables a more representative estimation of aberrant cVEMP results in the overall population of young hearing-impaired children. Recently, the above mentioned study of Verrecchia et al. showed a higher cVEMP abnormality rate of 36.4% in their small subgroup with a sensorineural hearing loss compared to our refer rate of 9.5%^47^. As already mentioned, this study also focussed on a different target population, including both normal-hearing and hearing-impaired infants. Moreover, the already discussed lower participation rate in this study may imply that parents, who were concerned about vestibular or motor deficiencies in their child, were more likely to participate, which may possibly lead to a higher percentage of vestibular (mainly saccular) dysfunction in the study sample^57^.

Regarding the relation with the laterality of hearing loss, the current study could not reveal a significantly different cVEMP refer rate in the group with a unilateral and bilateral sensorineural hearing loss, thereby highlighting the necessity of vestibular assessment even in infants with only unilateral hearing loss. These research findings are consistent with previous studies that also detected vestibular dysfunction in children with unilateral sensorineural hearing loss^58,59^. Concerning the relation with the degree of hearing loss, a significantly higher cVEMP refer rate occurred in infants with severe-profound compared to mild-moderate sensorineural hearing loss in the current study. In line with these results, Verbecque et al. reported higher percentages of deviant cVEMP results in more severely hearing-impaired children across literature^21^, which can be directly related to the close anatomical and embryological relationship of the cochlea and the otolith organs^21,36,37^.

The first limitation of this vestibular screening protocol is the use of only the cVEMP. As a result, the peripheral vestibular system is not completely evaluated, which may lead to an underestimation of the overall vestibular dysfunction in this population. The second limitation is that the vestibular function is screened only once at the age of 6 months, and in some cases twice if a retest is needed within 3 months. Therefore, children with a progressive or delayed-onset hearing or vestibular deficit will be missed as well. In spite of these two limitations, the VIS–Flanders project makes it possible to offer a basic vestibular screening to hearing-impaired infants on a large scale, as the cVEMP test is a short, objective and child-friendly test of which the equipment is widely available^33^. As longitudinal and extensive vestibular assessment is currently not feasible on a large scale in clinical practice, the vestibular screening at least ensures an early identification of vestibular (mainly saccular) dysfunction and subsequent motor referral if needed. Furthermore, the VIS–Flanders project increases awareness among parents and caregivers on vestibular dysfunction in hearing-impaired children, and provides information about the limitations of the screening test and the importance of an extensive vestibular assessment if necessary. In the future, it is aimed to more precisely map the predictive factors for vestibular dysfunction, such as the etiology of hearing loss, in a larger sample size of hearing-impaired infants in order to set guidelines to refine the vestibular screening protocol.

## Conclusion

The VIS–Flanders project is the first in its kind to implement a standard vestibular screening by means of the cVEMP test for hearing-impaired infants in an entire region. This study showed a 9.5% vestibular screening refer in a representative population of infants with unilateral or bilateral sensorineural hearing loss. This study also showed that the implementation of a vestibular screening programme is feasible, since a participation rate of 86.7% was achieved. In line with previous studies, a significantly higher proportion of infants with unilateral or bilateral severe-profound sensorineural hearing loss failed the vestibular screening compared to those with unilateral or bilateral mild-moderate sensorineural hearing loss (RR = 9.8). Besides the degree of hearing loss, future studies also need to focus on the underlying etiology of hearing loss in order to more precisely map the predictive factors for vestibular dysfunction in this population and to refine the vestibular screening protocol. As this study clearly indicates that a standard vestibular screening in hearing-impaired infants is feasible on a large regional scale, the VIS–Flanders project aims to increase awareness about vestibular dysfunction in hearing-impaired children by setting an example for other regions worldwide.

## Data Availability

As this study concerns a large-scale regional ongoing project and the dataset contains data of paediatric patients from all different participating centres, data will not be made available at this stage.
